# KNOWLEDGE AND PREDICTORS OF IMPLEMENTATION OF STANDARD PRECAUTIONS FOR INFECTION PREVENTION AND CONTROL AMONG HEALTH CARE PRACTITIONERS

**DOI:** 10.21010/Ajidv18i2S.5

**Published:** 2024-07-04

**Authors:** AYOGU Ebere Emilia, ARAFATI Aheebwa, EZEONWUMELU Joseph Obiezu, EBOSIE Jennifer, ISAH Abdulmuminu, GORUNTLA Narayana, UKWE Chinwe Victoria

**Affiliations:** 1 Department of Clinical Pharmacy and Pharmacy Practice, Kampala International University, School of Pharmacy, 00256, Western Campus, Ishaka, Uganda; 2 Department of Clinical Pharmacy and Pharmacy Management, Faculty of Pharmaceutical Sciences, University of Nigeria, Nsukka, Nigeria

**Keywords:** Knowledge, Implementation, Infection Prevention and Control, Uganda

## Abstract

**Background:**

Infection prevention and control involve health care practitioners, who are saddled with the duty of ensuring implementation of standard preventive measures to prevent healthcare associated infections.

**Objectives:**

To assess the knowledge and predictors of implementation of standard precautions for infection prevention and control among health care practitioners (HCPs).

**Material and method:**

A questionnaire-based cross-sectional design was employed in assessing HCPs in Uganda in from April – October 2023. Data were analyzed using descriptive, Pearsons’ correlation, linear and binary logistic regression with the aid of SPSS Version 22

**Results:**

Among the 222 healthcare practitioners assessed, 127 (57.2%) and 115 (51.8 %) had good knowledge and practice towards implementation of standard infection prevention and control precautions respectively. Chi square analysis indicated that age of healthcare practitioner (p=0.02; CI: 0.018 – 0.23), hospital unit of practice (p=0.003; CI: 0.002 – 0.004) and the type of facility where the health care practitioner works is significantly associated with their knowledge, while profession of the healthcare practitioner (p=0.002; CI: 0.001-0.003) and hospital unit of practice (p=0.002; CI: 0.001-0.003) were associated with implementation of the standard infection prevention and control precautions. Linear regression revealed knowledge is a significant predictor of good practice towards implementation of preventive measures (OR = 0.19; CI 0.102 – 0.272; p < 0.001).

**Conclusion.:**

Healthcare practitioners had poor knowledge and practice towards infection prevention. Thus, emphasizing continuous education and training for all healthcare professionals about infection prevention and control interventions as well as strict adherence to proper infection prevention and control practices.

## Introduction

According to the International Federation of Infection Control, infections have remained the biggest burden in health care service delivery, causing a major setback due to consequent increased health care costs (Nalunkuma *et al.*, 2021). A major setback is Health Care Acquired Infections (HCAIs), which is infection that occurs in a patient as a result of care at a health care facility and was not present at the time of arrival at the facility (Curless *et al.*, 2018). The US Centers for Disease Control and Prevention (CDC) also defines HCAIs as infections that begin either on or after day 3 of hospitalization (with the day of hospital admission being day 1) or on the day of discharge, or on the day after discharge (Curless *et al.*, 2018). The most common HCAIs of public health concern in many settings include, catheter-associated urinary tract infections, central line-associated bloodstream infection, surgical site infections, ventilator-associated pneumonia, multidrug-resistant infections, and infectious diarrhea and *Clostridium difficile* infections (Haque *et al.*, 2018).

Hospital acquired infection therefore has a major role to play in the prognosis and therapeutic outcome of both infectious or communicable diseases. Infection refers to the entrance of microorganisms (virus, bacteria, parasite or fungi) into the body leading to the body’s reaction while disease refers to any harmful deviation from the normal structural or functional state of an organism, generally associated with certain signs and symptoms indicative of its abnormal state and differing in nature from physical injury (Yongu, 2022). These can be communicable or non-communicable. Communicable diseases are infections caused by infectious agents, such as bacteria, viruses, parasites and can spread from one person to another e.g. Ebola, COVID-19, hepatitis, tuberculosis etc (WHO, 2019), while non communicable diseases are those that cannot be spread from one person to another.

Globally, 5–15% of hospitalized patients suffer from HCAIs and this is primarily due to poor infection prevention and control (IPC) practices in the hospitals (Opollo *et al.*, 2021) When extrapolating prevalence data to estimate the burden of HCAI on the healthcare system, it is estimated that over 2.6 million HCAI occur annually in the European Union (EU) countries. Further extrapolations suggest that these HAI account for a total of 501 disability-adjusted life years (DALYs) per 100,000 general populations and an attributable number of over 90,000 deaths per year (Aghdassi *et al.*, 2019). Although these figures majorly apply to the European context, various studies have illustrated that HCAI are also a problem in healthcare settings outside the EU, particularly low- and middle-income countries. In low-income and middle-income countries (LMICs), the frequency of HCAIs is estimated to be more than double compared to high-income countries (Opollo *et al.*, 2021).

Studies on spread of infectious diseases have analyzed the impact of infection prevention measures such as quarantine, isolation, contact tracing, and travel restrictions, on controlling the spread of communicable diseases and have found them to be essential in managing the afore mentioned outbreaks and preventing the transmission of Ebola and COVID 19 (WHO, 2019)

In response to the high spread of HCAIs, World Health Organization (WHO) has recommended several domains of infection prevention and control precautions to prevent and control the spread of infections in healthcare settings (WHO, 2019). Standard infection precautions are the minimum infection prevention practices that apply to all patient care, regardless of suspected or confirmed infection status of the patient. These form the standard precautions for the prevention and control of infections and they include: Patient assessment for infection risk, hand hygiene, respiratory and cough hygiene, personal protective equipment (PPE), safe management of equipment, safe management of environment, safe management of blood and body fluids and safe management of linen. Standard precautions are sets of recommendations designed to prevent or minimise exposure to infectious agents by hospital staff, patients and their visitors (Ogoina *et al.*, 2015).

Healthcare practitioners (HCPs) are pivotal in the implementation of these infection prevention and control measures. Their role in the spread and control of HAIs cannot be overemphasized. In the course of health service delivery, healthcare practitioners are themselves often exposed to infections and other transmissible disease occurring within the hospital setting. HCPs are not only at risk of acquiring infections but also of being a source of infection to patients. Therefore, both the patient and the HCP need to be protected from contracting or transmitting hospital-acquired infections by using recommended infection control measures (International Society for infectious disease, 2023). The safety of HCPs is even of more clinical significance than that of the patient as prevention of infectious diseases in HCPs benefits the healthcare system in three ways: preservation of the health of the HCPs, prevention of work restrictions, and the reduction of hospital-acquired infections (Margreet, 2018). Globally, standard precautions of infection control are considered as effective means of protecting healthcare workers, patients and the public and reducing nosocomial infections.

It is paramount to emphasize that education and knowledge is an important factor for improving compliance with guidelines and prevention measures. All HCPs, as a matter of necessity, need to be knowledgeable on the standard infection prevention and control measures in order to enable their effective implementation.

Increase in the prevalence of healthcare acquired infections has been reported in Uganda (Greco and Magombe, 2011), with infectious diseases accounting for 18% of all hospital deaths and 37% of hospital admissions (Federal Ministry of Health Uganda, 2018). Amidst this unpleasant situation, an overall Infection Prevention and Control compliance score at some health facility was 225/800 (28.5%), (Opollo *et al.*, 2021). Consequent to this development, the outbreak of COVID-19 and Ebola, coupled with high mortality and morbidity rates due to HCAIs, Ugandan Ministry of Health developed policies and guidelines aimed at preventing and minimizing the risk of healthcare acquired infection within the community and the health facilities. These were expected to be implemented throughout the country and implementation was to be monitored by an Infection Prevention and Control committee at a national level and in every tertiary health facility (Opollo *et al.*, 2021). However, how knowledgeable the healthcare practitioners are with regards to these standard precautions for infection prevention and control and how successful they have been implemented within Western Uganda still remains unknown. Hence, this study seeks to assess the knowledge and implementation of standard precautions of infection prevention and control among healthcare practitioners at different health center levels in Bushenyi district, Western Uganda

## Materials and Methods

### Study design and site.

A cross-sectional study was used to assess the knowledge and implementation of standard precautions for infection prevention and control among healthcare practitioners working at different health center levels at Bushenyi District located in Western Uganda. Uganda is a land-locked East African country with a population of 41.6 million in June 2020 (Uganda Bureau of Statistics, 2020). It has 4 geopolitical zones namely, Central, Western, Eastern, and Northern regions. Health wise, there are 17 Regional Referral Hospitals (RRHs) and 62 are General Hospitals (GHs) and a network of primary health centres in the country (Ugandan Bureau of Statistics, 2020). Uganda’s health facilities are classified into seven levels based on the services they provide and the catchment area they are intended to serve. The health facilities are designated as Health Centre level one (HC I) to Health Centre Level four (HC IV); General hospital, Regional Referral hospital and National Referral hospital.

Bushenyi District has three (3) district hospitals, two (2) health center IV, eleven (11) health center III, and sixteen (16) health center II. According to Uganda health system, health center II provides mainly basic out-patient health services, health education, primary health care, HIV testing and counselling, family planning services, dispensing of drugs that manage common conditions such as cough, flue, malaria and is headed by a nursing officer. Health Centre III provide all services offered by health center II and an additional maternity service, laboratory services and headed by a Clinical Officer. Health Centre IV provide all services provided by Health Centre III with additional dental, surgery, ART services and are headed by general medical doctor. Hospitals provide all activities offered by health center IV with specialized health care services and is headed by a Medical Superintendent.

### Study population and sample size determination

The health facilities were classified into tertiary, secondary and primary based on the type of services they rendered. The study included health facilities from facilities two (2) tertiary, nine (9) secondary and twelve (12) primary facilities.

**Tertiary health facilities** included were Kampala International Teaching Hospital Western Campus, and Adventist Specialist Hospital;

**Secondary health facilities** included were three (3) health center IV and six (6) health center III namely Bushenyi Health Centre IV, Bushenyi Medical Centre and Kyabugimbi Health Centre IV; namely Kyamuhunga Health Centre III, Kashambya Health Centre III, Bitooma Health Centre III, Kyeizooba Health Centre III, Nyamiko Health Centre III, Ruhumuro Health Centre III.

**Primary health facilities** are health center II namely Kyeizooba Farmers Clinic, Reproductive Health Uganda, Uganda Prison Health Centre II, Uganda Police Health Centre, Buyanja Health Centre II, Nyamyaga Health Centre II, Bwera Health Centre II, Ruharo Health Centre II, Kashoshoga Health Centre II, Rutooma Health Centre II and Kajunju Health Centre II.

Sample size was calculated using Raosoft sample size calculator (http://www.raosoft.com/samplesize.html) setting margin of error at 5%, confidence level at 95%, population size of 500 and response distribution at 50%. The sample size was calculated to be 218. A simple random sampling technique was employed to select the representative subjects from all healthcare providers list in Bushenyi district.

### Eligibility Criteria

This study included all health workers who are fully employed at the selected health centers within Bushenyi district and gave their consent to participate. Health workers who were part time staff and did not consent were excluded.

### Data collection tool

A structured questionnaire was developed and used to collect the data by the researcher and the research assistants. The questionnaire consists of three sections; section one assessed practitioners’ demographic characteristics. It has 5 questions on gender, age, profession, hospital unit, years of practice and the type of facility. Section 2 has 21 questions that assessed their knowledge on definition of SPIPC, components of SPIPC and measures taken in the event of exposure to body fluid or blood while section 3 has 11 questions that assessed their practice towards hand hygiene, use of personal protective gears, actions taken in event of exposure to blood and body fluids and institution of committee for monitoring SPIPC.

### Questionnaire development

Sets of questions assessing knowledge and practices towards implementation, were developed from the WHO guideline for standard precaution for infection prevention and control (SPIPC). The questions were all close ended. Quality control measures were put in place to ensure validity and reliability of the questionnaire. This was done in 3 phases.

### Phase 1: Content validity

This initial questionnaire was sent to three (3) professionals in the area of disease and infection control for content validation. They assessed the potentials of the question to assess health workers’ knowledge on SPIPC and their practices towards its implementation. Their comments and inputs were adequately followed in reviewing the questionnaire for the second phase of validation.

### Phase 2: Face validation

The final version of the questionnaires was distributed among 20 health care practitioners not intended to participate in the research or from the selected facilities. This was to assess the overall validity, clarity and completeness of the questions. All information and comment gathered were used to finally edit the questionnaire to the final version used.

### Phase 3: Questionnaire reliability testing

The questions were tested for consistency or the degree to which they elicited the same kind of information each time they were asked. This was done by administering the content validated questionnaires to 20 volunteer participants for pre-testing. Their responses to each question were recorded and reliability analysis was done. An acceptable Cronbach alpha value of 0.75 was obtained for the questionnaire.

### Data Analysis

Data were analyzed using the Statistical Package for Social Sciences (SPSS) version 22. Descriptive statistics (frequencies and percentages) were used to summarize the healthcare practitioners’ demographic characteristics. Knowledge and practice questions were restructured into “Yes” and “No” responses, where correct answers were assigned 1 and wrong answers assigned 0. Health practitioners with values above or below the median value were categorize as having “Good” or “Poor” knowledge and practice respectively. Pearson Chi square was used to determine the association between the healthcare practitioners’ demographic characteristics and their level of sum knowledge and Practice scores. Pearsons’ correlation was used to assess the relationship between respondents’ knowledge and their practice towards implementation of the standard precaution for infection prevention and control measures. Linear regression was used to determine whether knowledge is a predictor for good practice towards implementation of infection control measures while binary logistic regression was used to determine which demographic characteristics predicts good practices towards implementation of the standard precaution for infection prevention and control measures among the healthcare practitioners. Difference in variables were considered statistically significant at p < 0.05 and confidence interval of 95%. For percentages less than 5%, Monte Carlo Significant values were used instead of Chi square values.

### Operational definitions

In this study, consultant physicians are medical personnels who have specialized in a particular aspect of internal medicine. Medical officers are medical personnels who are general practitioners while clinicians are those who obtained diploma in clinical and community medicine.

### Ethical considerations

Ethical approval of the study was sought and obtained from the Research and Ethics Committee (REC) of Kampala International University - KIU-REC/2023/PP/017. Permission to assess staff of each facility was gotten from the officer in-charge of every health facility used while informed and written consents were also obtained from every participant before enrolment. All obtained data for this research were handled in accordance with relevant guidelines (Declaration of Helsinki 2013).

## Results

### Socio demographics data of the health care practitioners.

The results show the characteristics of 222 respondents. Of those surveyed, (51.8%) were male and (48.2%) were female. The majority of the respondents were aged between 20-30 years (44.1%) followed by 31-40 years (41.4%). Nurses represented the largest professional group (58.1%) followed by clinicians 37 (16.7%). The most common department of work was outpatient (43.2%) followed by laboratory (15.8%) and most healthcare workers had been practicing for 1-5 years (41.0%). The majority of the healthcare workers (59.9%) worked in secondary facilities. Majority of the respondents 219 (98.6%) knew of IPC. (Table 1).

**Table 1:** Socio demographic characteristics of the healthcare practitioners.

Characteristic	Category	Frequency (%) N = 222
**Gender**	Male	115(51.8)
	Female	107(48.2)
**Age**	<20 years	4(1.8)
	20-30 years	98(44.1)
	31-40 years	92(41.4)
	41-50 years	20(9.0)
	>50 years	8(3.6)
**Profession**	Consultant physician	3(1.4)
	Medical doctor	14(6.3)
	Clinician	37(16.7)
	Pharmacist	5(2.3)
	Laboratory scientist	5(2.3)
	Laboratory Technician	26(11.7)
	Nurse	129(58.1)
	Health assistant	3(1.4)
**Hospital Unit**	Medical	27(12.2)
	Ophthalmology	3(1.4)
	Laboratory	35(15.8)
	Accident and Emergency	16(7.2)
	Surgical unit	8(3.6)
	Pharmacy	12(5.4)
	Obstetrics and Gynaecology	25(11.3)
	Out patient	96(43.2)
**Years of practice**	Below 1	9(4.1)
	1-5	91(41.0)
	6-10	70(31.5)
	11-20	43(19.5)
	>20	9(4.1)
Type of facility*	Primary	33(14.9)
	Secondary	133(59.9)
	Tertiary	56(25.2)

* Primary – Health center II; Secondary – Health III and IV; Tertiary – Hospitals

### Health care practitioners’ knowledge about the standard infection prevention and control precautions.

Participants were assessed on their knowledge about SIPC. Statistical analysis of their responses showed that 127 (57.2%) of the health care practitioners had good knowledge of the standard infection prevention and control precautions while 95 (42.8%) had poor knowledge. A total of 189(85.1%) knew the correct definition of standard infection prevention and control precautions, 140 (63.1%) knew about Aide-Memoire as a document containing guidance statements providing a platform to ensure that guidelines are applied in practice and 182 (82.0) knew that there is a need to change pair of gloves between patients even when there is no visible dirt or contamination**.** Other knowledge questions and their responses are displayed in Table 2. Chi square analysis indicated that age of healthcare practitioner (p=0.02; CI: 0.018 – 0.23), hospital unit of practice (p=0.003; CI: 0.002 – 0.004) and the type of facility where the health care practitioner works is significantly associated with their knowledge about association test between the standard infection prevention and control precautions as shown in Table 3.

**Table 2a:** Knowledge on the standard infection prevention and control precautions among health care workers

S/no	Questions	Responses	Frequency (%) N=222
1	SPIPC is the use of hand sanitizer and face mask for infection control	Correct	217 (97.7)
		Incorrect	5 (2.3)
2	SPIPC is the evidence based-practical approach to treatment of infections in patients	Correct	217 (97.7)
		Incorrect	5 (2.3)
3	SPIPC is the evidence-based practices and procedure for the prevention and reduction of transmission of microbes to health care workers, patients and visitors	Correct	189 (85.1)
		Incorrect	33 (14.9)
4	SPIPC is the practices and procedures applied for the prevention of infection transmission in patients	Correct	195 (87.8)
		Incorrect	27 (12.2)
5	Does standard infection control precautions involve patient assessment for infection risk?	Correct	93 (41.9)
		Incorrect	129 (58.1)
6	Does standard infection control involve control precautions involving respiratory and cough hygiene?	Correct	79 (35.6)
		Incorrect	143 (64.4)
7	Does standard infection control precautions involve safe management of equipment?	Correct	139 (62.6)
		Incorrect	83 (37.4)
8	Does standard infection control precautions involve safe management of blood and body fluids?	Correct	113 (50.4)
		Incorrect	109 (49.1)
9	Does standard infection control precautions involve hand hygiene?	Correct	189 (85.1)
		Incorrect	33 (14.9)
10	Does standard infection control precautions involve use of personal protective equipment?	Correct	187 (84.2)
		Incorrect	35 (15.8)
11	Does standard infection control precautions involve safe management of the environment?	Correct	87 (39.2)
		Incorrect	135 (60.8)

**Table 2a:** Knowledge on the standard infection prevention and control precautions among health care workers

S/no	Questions	Responses	Frequency (%) N=222
12	Does standard infection control precautions involve safe management of linen?	Correct	142 (64.0)
		Incorrect	80 (36.0)
13	Aide-Memoire is a document containing guidance statements providing a platform to ensure that guidelines are applied in practice	Correct	140 (63.1)
		Incorrect	82 (36.9)
14	Does wearing gloves eliminate the need for hand washing?	Correct	156(70.3)
		Incorrect	66 (29.7)
15	Is the use of 70% alcohol- based antiseptic as effective as Soap and water in infection control?	Correct	151 (68.0)
		Incorrect	71 (32.0)
16	Is there a need to change pair of gloves between patients as long as there is no visible dirt or contamination?	Correct	182 (82.0)
		Incorrect	40 (18.0)
17	Should sodium hypo chloride 0.5% bleach be used to clean up spills?	Correct	158 (71.6)
		Incorrect	63 (28.4)
18	In case of injury involving blood, washing wound with soap and water is one of the best steps recommended	Correct	82 (36.9)
		Incorrect	140 (63)
19	In case of injury involving blood, irrigating eyes with water, saline or sterile irrigants is one of the steps recommended	Correct	100 (45.0)
		Incorrect	122 (55.0)
20	In case of injury involving blood, immediate seek of medical treatment is one of the steps recommended	Correct	162 (73.0)
		Incorrect	60 (27.0)
21	In case of injury involving blood, flushing out mouth, nose or skin with water is one of the steps recommended	Correct	100 (45.0)
		Incorrect	122 (55.0)
22	In case of injury involving blood, reporting the incident to your supervisor is one of the steps recommended	Correct	127 (57.2)
		Incorrect	95 (42.8)

SPIPC – Standard precautions for infection prevention and control

**Table 3:** Association between the demographic characteristics of respondent and level of Knowledge on infection prevention and control interventions.

Characteristics	Category	Poor knowledge (%)	Good knowledge (%)	P value	95% Confidence Interval
Gender	Male	52 (45.2)	63 (54.8)	0.50	0.491- 0.511
	Female	43 (40.2)	64 (59.8)		
Age (Years)	<20	2(50.0)	2(50.0)	0.02	0.018 – 0.23
	20-30	48 (49.0)	50 (51.0)		
	31-40	28 (30.4)	64 (69.6)		
	41-50	12 (60.0)	8 (40.0)		
	>50	5 (62.5)	3 (37.5)		
Profession	Consultant physician	1 (33.3)	2(66.7)	0.38	0.380 – 0.399
	Medical doctor	3 (21.4)	11 (78.6)		
	Clinician	15 (40.5)	22 (59.5)		
	Pharmacist	2 (40.0)	3 (60.0)		
	Lab. Scientist	4 (80.0)	1 (20.0)		
	Lab. Technician	9 (34.6)	17 (65.4)		
	Nurse	59 (45.7)	70 (54.3)		
	Health Assistant	2 (66.7)	1 (33.3)		
Unit /section	Medical	10(37.0)	17(63.0)	0.003	0.002 – 0.005
	Ophthalmology	1(33.3)	2(66.6)		
	Laboratory	15(42.9)	20(57.1)		
	Accident and Emergency	14(87.5)	2(12.5)		
	Surgical unit	2(25.0)	6(75.0)		
	Pharmacy	6(50)	6(50)		
	Obstetrics and Gynecology	5(20.0)	20(80)		
	Out patient	42(43.8	54(56.9)		
Years of practice	Below 1 year	6_ (_66.7)	3 (33.3)	0.30	0.300 – 0.318
	1-5	39 (42.9)	52 (57.1)		
	6-10	27 (38.6)	43 (61.4)		
	11-20	17 (39.5)	26 (60.5)		
	> 20	6 (66.7)	3 (33.3)		
Type of facility	Primary level	17 (51.5)	16 (48.5)	0.003	0.002 – 0.004
	Secondary level	45 (33.8)	88 (66.2)		
	Tertiary level	33 (58.9)	23 (41.1)		

*Primary – Health center II; Secondary – Health III and IV; Tertiary – Hospitals

### Practices towards implementation of standard infection prevention and control precautions among the health care workers

Among the assessed health care practitioners, 115 (51.8 %) had good practice while 107 (48.2 %) had poor practice towards implementation of standard infection prevention and control precautions. The results of this study showed that 146 (65.8%) of respondents always wash their hands with proper detergents before patient contact, while 183 (82.4%) use hand sanitizer or hand rub to clean hands. Additionally, 118 (53.2%) of respondents always use personal protective gear to prevent infection transmission, 60% of respondents would take post-exposure prophylaxis if exposed to blood, body fluids, or needle prick injury as shown in Table 4. Chi square analysis showed that profession of the healthcare practitioner (p=0.002; CI: 0.001-0.003) and hospital unit of practice (p=0.002; CI: 0.001-0.003) were significantly associated with implementation of the standard infection prevention and control precautions as displayed in Table 5.

**Table 4:** Practice towards implementation of standard infection prevention and control precautions among the health care practitioners

S/No	QUESTION	RESPONSE	FREQUENCY(%)
**1**	How often do you wash hands with proper detergents before patient contact?	Never	23(10.4)
		Some times	53(23.9)
		Always	146(65.8)
**2**	Do you always use antiseptics hand sanitizer to clean hands?	Yes	183(82.4)
		No	36(16.2)
**3**	Have you ever been exposed to blood or other body fluid of patients through contact or unprotected skin?	Never	148(66.7)
		Sometimes	70(31.5)
		Always	4(1.8)
**4**	How often do you use personal protective gears to prevent infection transmission	Never	21(9.5)
		Sometimes	82(36.9)
		Always	118(53.2)
**5**	In the case of exposure to blood, body fluids or needle prick injury, which action would you take?		
	Taking post exposure prophylaxis only	Yes	80(60)
	No	142(40)
	Cleaning affected area with water and antiseptic only	Yes	25(84.2)
	No	197(15.8)
	Both actions no 5 and 6 would be a measure taken	Yes	119(53.6)
	No	103(46.4)
	Not sure of the measure to be taken	Yes	27(12.2)
	No	195(87.8)
**6**	How often does your hospital procure and distribute tissues, medical masks and alcohol- based hand rub?	Never	28(12.6)
		Sometimes	37(16.7)
		Always	157(70.7)
**7**	How often do you access the availability and use of personal protective equipment in the patient care unit?	Never	20 (9.0)
		Sometimes	44 (19.8)
		Always	158 (71.2)
**8**	How often do you have access to running water?	Never	27 (12.2)
		Sometimes	36 (16.2)
		Always	159 (71.6)
**9**	Does your facility have a committee monitoring infection prevention and control intervention practices?	Yes	156(29.7)
		No	66(70.3)
**10**	If yes, do they give the administration targeted feedback?	Yes	82(37)
		No	140(63)

**Table 5:** Association between respondents’ demographic characteristics and practice of standard infection prevention and control precautions.

Characteristics	Category	Poor knowledge (%)	Good knowledge (%)	P value	95% Confidence Interval
Gender	Male	50(43.5)	65(56.5)	0.17	0.171-0.186
	Female	57(53.3)	50(46.7)		
Age (Years)	<20	1(25)	3(75)	0.354	0.344-0.363
	20-30	43(43.9)	55(56.1)		
	31-40	48(52.2)	44(47.8)		
	41-50	9(45.0)	11(55)		
	>50	6(75)	2(25.0)		
Profession	Consultant physician	1(33.0)	2(66.7)	0.002	0.001-0.003
	Medical doctor	3(21.4)	11(78.6)		
	Clinician	22(59.5)	15(40.5)		
	Pharmacist	1(20)	4(80)		
	Laboratory Scientist	2(40)	3(60)		
	Laboratory Technician	5(19.2)	21(80.8)		
	Nurse	72(55.8)	57(44.2)		
	Health Assistant	1(33.3)	2(66.7)		
Unit /section	Medical	11(40.7)	16(59.3)	0.002	0.001-0.003
	Ophthalmology	2(66.7)	1(33.3)		
	Laboratory	8(22.9)	27(77.1)		
	Accident and Emergency	5(31.3)	11(68.8)		
	Surgical unit	4(50)	4(50)		
	Pharmacy	4(33.3)	8(66.7)		
	Obstetrics and Gynecology	13(52.0)	12(48.0)		
	Out patient	60(62.5)	36(37.5)		
Years of practice	Below 1 year	4(44.4)	5(55.5)	0.987	0.985-0.989
	1-5	44(48.4)	47(51.6)		
	6-10	34(48.6)	36(51.4)		
	11-20	20(46.5)	23(53.5)		
	> 20	5(55.5)	4(44.4)		
Type of facility	Primary level	20(60.6)	13(39.4)	0.213	0.204-0.221
	Secondary level	64(48.1)	69(51.9)		
	Tertiary level	23(41.1)	33(58.9)		

*Primary – Health center II; Secondary – Health III and IV; Tertiary – Hospitals

To assess the relationship between HCPs knowledge and their practice, Pearson’s correlation analysis was done. The result showed a positive significant but weak relationship between knowledge and practice (r = 0.150; *P* = 0.026).

### Determination of predictors of good practice towards implementation of standard infection prevention and control precautions

Linear regression analysis revealed that knowledge is a significant negative predictor of good practice towards implementation of preventive measures (OR = 0.19; CI 0.102 – 0.272; p < 0.001), while binary logistic regression indicated that none of the demographics characteristics was a predictor of good practice towards implementation of standard infection prevention and control precautions as shown in Table 6.

**Table 6:** Binary logistic regression analysis of demographic characteristics and implementation of standard infection prevention and control precautions.

Characteristics	Categories	OR (95%CI)	p-value
Age (years)	<20	Ref	.236
	20-30	.349 (027 - 4.530)	.421
	31-40	.208 (.015 - 2.805)	.237
	41-50	.319 (.019 - 5.487	.431
	>50	.052 (.002 - 1.293)	.071
Profession	Consultant physician	Ref	.494
	Medical doctor	2.055 (.082 - 51.250)	.661
	Clinician	.442 (.023 - 8.655)	.591
	Pharmacist	1.594 (.030 - 84.783)	.818
	Lab scientist	.323 (011 - 9.660)	.514
	Lab. Technician	1.144 (.060 - 21.810	.929
	Nurse	.481 (.025 - 9.376	.629
	Health assistant	1.571(.034 - 71.659)	.817
Hospital Unit	Medical	Ref	.460
	Ophthalmology	.373 (.026 - 5.354)	.468
	Laboratory	1.586 (.134 - 18.798)	.715
	Accident and Emergency	1.884 (.415 - 8.558)	.412
	Surgical unit	.526 (.086 - 3.227)	.487
	Pharmacy	1.068 (.184 - 6.197)	.941
	Obstetrics and Gynecology	.621 (.184 – 2.094)	.442
	Out patient	.455 (.174 - 1.192)	.109
Years of practice	Below 1	Ref	.829
	1-5	.827 (.165 - 4.135)	.817
	6-10	1.100 (.209 - 5.785)	.910
	11-20	1.525 (.269 - 8.637)	.633
	>20	1.177 (.107 - 12.893)	.894
Type of facility	Primary level	Ref	.911
	Secondary level	1.107 (.468 - 2.622)	.817
	Tertiary level	.922 (.291 - 2.915)	.890

*Primary – Health center II; Secondary – Health III and IV; Tertiary – Hospitals

## Discussion

In this study, the knowledge and practice of healthcare practitioners towards implementation of infection precaution and prevention control were assessed and our findings revealed that just about half of the healthcare practitioners had good knowledge and good practice. A major consequence of poor knowledge and practice towards implementation of SPIPC is increased healthcare-associated infections. According to the World Health Organization fact sheet, (2019), HCAI reported that a hundred million patients were affected each year globally. This has necessitated the development of these standard precautions for infection prevention and control, which has a greater percentage of its implementation domicile among healthcare practitioners.

### Socio demographic characteristics of the healthcare workers.

In this study, about half of the respondents were within 20-40 years which is similar to a report from North west Ethiopia (Desta *et al.*, 2018). This supports the report by WHO which stated that in the recent times, the world has more young people than ever before, in the global work force (WHO, 2020). Nurses constituted half of the entire participants, which is also in tandem with a study carried out in Nigeria (Iliyasu *et al.*, 2016). Most of the healthcare workers had 1-5 years of work experience while one third had 6-10 years of work experience. This is in agreement with what was obtainable in Ethiopia, where about 74% had work experience of less than 5 years (Desta *et al.*, 2019). This may be attributed to the need or desire for a better opportunity among youths, who are faced with multifaceted challenges, however this practice will have a negative impact in infection control as such instability among HCP does not encourage increase in knowledge and perfection of acquired skills.

### Assessing the level of knowledge about the standard precautions for infection prevention and control precautions.

Only half of the HCP were had good knowledge of standard infection prevention and control precautions, which is lower than 67.7% recorded in Quassim, Saudi Arabia (Abalkhail *et al.*, 2021), 70.8% in Northeast Ethiopia and 84.7% in Northwest Ethiopia (Desta *et al.*, 2019). This higher poor knowledge observed in Uganda compared to other countries could be attributed to inadequate training among HCPs, high level of poverty and poor healthcare service. Majority of them knew the correct definition of standard precautions for infection prevention and control but were not knowledgeable about the component of the preventive measures to be taken to avoid onset of infection. More than half of them did not know that these measures involve respiratory and cough hygiene and patient assessment for infection risk, management of blood and body fluids, safe management of the environment etc. The implication is that they are likely to be more effective in infection control when there is an outbreak already than in preventing the outbreak of diseases. Healthcare workers within the age of 31-40 had the highest knowledge score among others, while the medical officers were the most knowledgeable among health professionals. This finding differs from that of Illiyasu *et al*, (2016) which reported that nurses in Northwest Nigeria were more knowledgeable compared to other HCPs.

In terms of unit, the obstetrics and gynecology and the surgical unit had better knowledge compared to other units, this could be due to the fact that they are dealing with aseptic conditions that require sterile environment. The HCPs with 6-10 years of work experience had the highest good knowledge score compared to others, surprisingly, those with less than one year and over 20 years working experience had the least knowledge. This observation could be understandable in HCPs with less than one year work experience as they may not have had encounter, however the poor knowledge among those of over 20 years was not expected. The possible reason for this could be a false over-familiarity with hospital environment and disease states and fatigue towards keeping up with instructions.

The demographic characteristics and level of knowledge indicate that age of the practitioner (p=0.02, CI: 0.018 – 0.23), unit or section of service delivery in the facility (p=0.003, CI: 0.002 – 0.005) and type of facility of practice (p=0.003, CI: 0.002 – 0.005) were all significantly associated with knowledge level of the health practitioners, while year of practice and profession were not associated with knowledge. This implies that knowledge about the standard infection, prevention and control precautions could be affected by the age of healthcare practitioners, hospital unit or section and type of facility the healthcare practitioner worked. These findings agree with a study on knowledge and practice of infection prevention and control among health workers in Northwest Ethiopia that reported association between knowledge and age of HCPs (Desta *et al.*, 2019)

### Practice towards implementation of standard infection prevention and control precautions among health care workers at different health center levels in Bushenyi district.

About half of the healthcare professionals had good practice towards implementation of precautions for infection prevention and control. This is poor compared to other studies carried out in Saudi Arabia, Northeast Ethiopia, and Northwest Ethiopia which reported better practice towards implementation of IPC measures [Desta *et al.*, 2019; Assefa *et al.*, 2020; Abalkhail *et al.*, 2021). In terms of hand hygiene, regular washing of hands is about the least of all precaution measures and a 100% compliance is expected in any health facility. In this study, even though majority of the HCPs used hand sanitizer, only one third of them washed their hands always portraying a poor practice compared to a study from Nigeria where 95.7 % of them washed their hands always (Orji *et al.*, 2023). This poor practice towards hand hygiene may be attributed to poor access to running water in the facility. One third of them have also been exposed to blood when they were unprotected. This implies that in practice these healthcare practitioners may not only serve as medium for infection transmission from patient to patient but are also highly vulnerable to being infected themselves. To ensure strict adherence to IPC measures, WHO released the Infection Prevention and Control Assessment Framework (IPCAF) in 2018, which enables healthcare facilities to monitor and evaluate IPC structures and practices (Aghdassi *et al.*, 2020). However, two third admitted that their facilities do not have a committee for monitoring the implementation of these infection prevention and control measures, while among those that have such committee, about two third of them do not give feedback on their findings. These findings indicate poor follow-up and monitoring of these preventive and control measures leading to poor infection control and increasing incidence of healthcare acquired infection. This poor practice towards assessment and evaluation of implementation practices could be attributed to poor awareness of such assessment framework tool by the health facility mangement

The level of good knowledge observed in this study among the HCPs did not translate to good practice. HCPs below the age of 20 years had the best practice compared to their older counterparts. The medical officers had the highest knowledge but the laboratory technicians and pharmacists had the best practices. HCPs with 11-20 years of work experience had the highest good practices while against the secondary facility that had the highest knowledge, the tertiary facility had the best practices towards implementation of IPC measures.

Statistical analysis revealed a significant association between profession of practitioner (p=0.02, CI: 0.001 – 0.003) and hospital unit or section practice (p=0.02, CI: 0.001 – 0.003) towards implementation of standard precautions for infection prevention and control. However, there was no relationship between type of facility and practice of standard infection prevention and control precautions. This finding does not agree with the work of Desta *et al.*, (2019) which reported association between knowledge of the HCPs and their age, work experience and education.

### Implications of the study findings for healthcare policy and practice

The findings of this work have identified some knowledge gap among the HCPs depicting the need for a focused and radical educational/training in these areas. In terms of knowledge, the area of emphasis is majorly in the knowledge about infection prevention and not control; focusing on the components of infection prevention. Such components like patient assessment for infection risk, respiratory and cough hygiene, safe management of equipment, environment, blood and body fluids should be given attention when developing curriculum for the health education. For practice, policies that will enforce good hand hygiene and establishment of IPCP monitoring committee should be established. Identified deficiencies in their existing practices are likely to be due to lack of monitoring and follow up, hence it can be improved by direct observation and closed-circuit television monitoring.

### Determination of predictors of good practice towards implementation of standard infection prevention and control precautions

Pearson’s correlation analysis showed a positive significant but weak relationship between knowledge and practice (r = 0.150; *P* = 0.026) this implies that increase in knowledge among healthcare practitioners will result in increase in good practice. Linear regression analysis revealed that knowledge is a significant negative predictor of good practice towards implementation of preventive measures as a 0.19 increase in knowledge will lead to a unit decrease in practice. (OR = 0.19; CI 0.102 – 0.272; *P* < 0.001). This analysis is supported by the practice observed among the HCPs which was not commensurate to the knowledge displayed by them. This observation was not expected as naturally, increase in knowledge should translate to increased practice. The possible reason for this anomaly was not clearly understood. Binary logistic regression indicated that none of the demographics characteristics was a predictor of good practice towards implementation of standard infection prevention and control precautions. This finding also differs from the work by Abalkhail *et al*, (2021) that age of HCPs and years of work experience were predictors of good practice.

### Limitations to the study

It is expedient to state that knowledge and implementation observed may differ after a period of time as the study employed a cross-sectional study which may not/cannot represent behavior over a period of time. There is need therefore for a longitudinal study to ascertain the practice over a period of time.

## Conclusion

The percentage of healthcare professionals having poor knowledge of standard infection prevention and control precautions, highlights the need for continuous training and education on infection prevention and control practices, as well as monitoring of compliance with these practices among health care practices.

Therefore, these results emphasize continuous education and training for all healthcare professionals and development and implementation of policies that will enhance infection control, by the government through their Federal Ministry of Health.

### Authors’ contribution

EEA, CVU and AA contributed to the conception and design of the work, while JE and AI, contributed substantially to the acquisition and analysis of data for the work. EEA, AI and JOE developed the theoretical formalism for this work and critically revised the drafted manuscript for important intellectual content. AA, EEA and NG drafted the manuscript while NG and CVU approved of the final version to be published. All authors read the final version and agreed that the manuscript is a true representation of the research work carried out.

**List of abbreviations:**
IPC: Infection Prevention and Control:
HCPs: Health Care Practitioners:
CDC; Disease Control and Prevention
EU: European Union:
RRHs: Regional Referral Hospitals:
HCI-IV: Health Center 1-4:
HCAIs: Health Care Acquired Infections:
LMICs: Low-income and middle-income countries:
SPIPC: Standard Precaution for Infection Prevention and Control:
PPE: Personal Protective Equipment:
IPCAF: Infection Prevention and Control Assessment Framework
